# Died

**Published:** 1862-12

**Authors:** 


DIED

SUDDENLY on the morning of November the 29th, EMMOR TAFT, student of Dental Surgery, and second son of Prof. J. Taft, in the eighteenth year of his age.

Not here and now do we speak of the crushing sorrow of his parents and friends. That is sacred. Let not cool calculating professional thoughts intrude themselves there. But, as a profession, we may lament the loss of the early dead more than we mourn the departure of those matured. The true professional man could not fail to regard Emmor as a bud of promise to his profession and to the cause of science; but his bloom is not here. W.

